# Small-scale mutations are infrequent as mechanisms of resistance in post-PARP inhibitor tumour samples in high grade serous ovarian cancer

**DOI:** 10.1038/s41598-023-48153-x

**Published:** 2023-12-11

**Authors:** Nikki L. Burdett, Madelynne O. Willis, Ahwan Pandey, Sian Fereday, AOCS Study Group, D. Bowtell, G. Chenevix-Trench, A. Green, P. Webb, A. DeFazio, D. Gertig, N. Traficante, S. Fereday, S. Moore, J. Hung, K. Harrap, T. Sadkowsky, N. Pandeya, M. Malt, A. Mellon, R. Robertson, T. Vanden Bergh, M. Jones, P. Mackenzie, J. Maidens, K. Nattress, Y. E. Chiew, A. Stenlake, H. Sullivan, B. Alexander, P. Ashover, S. Brown, T. Corrish, L. Green, L. Jackman, K. Ferguson, K. Martin, A. Martyn, B. Ranieri, J. White, V. Jayde, P. Mamers, L. Bowes, L. Galletta, D. Giles, J. Hendley, K. Alsop, T. Schmidt, H. Shirley, C. Ball, C. Young, S. Viduka, Hoa Tran, Sanela Bilic, Lydia Glavinas, Julia Brooks, R. Stuart-Harris, F. Kirsten, J. Rutovitz, P. Clingan, A. Glasgow, A. Proietto, S. Braye, G. Otton, J. Shannon, T. Bonaventura, J. Stewart, S. Begbie, M. Friedlander, D. Bell, S. Baron-Hay, A. Ferrier, G. Gard, D. Nevell, N. Pavlakis, S. Valmadre, B. Young, C. Camaris, R. Crouch, L. Edwards, N. Hacker, D. Marsden, G. Robertson, P. Beale, J. Beith, J. Carter, C. Dalrymple, R. Houghton, P. Russell, M. Links, J. Grygiel, J. Hill, A. Brand, K. Byth, R. Jaworski, P. Harnett, R. Sharma, G. Wain, B. Ward, D. Papadimos, A. Crandon, M. Cummings, K. Horwood, A. Obermair, L. Perrin, D. Wyld, J. Nicklin, M. Davy, M. K. Oehler, C. Hall, T. Dodd, T. Healy, K. Pittman, D. Henderson, J. Miller, J. Pierdes, P. Blomfield, D. Challis, R. McIntosh, A. Parker, B. Brown, R. Rome, D. Allen, P. Grant, S. Hyde, R. Laurie, M. Robbie, D. Healy, T. Jobling, T. Manolitsas, J. McNealage, P. Rogers, B. Susil, E. Sumithran, I. Simpson, L. Mileshkin, G. Au-Yeung, K. Phillips, D. Rischin, S. Fox, D. Johnson, S. Lade, M. Loughrey, N. O’Callaghan, W. Murray, P. Waring, V. Billson, J. Pyman, D. Neesham, M. Quinn, C. Underhill, R. Bell, L. F. Ng, R. Blum, V. Ganju, I. Hammond, Y. Leung, A. McCartney, M. Buck, I. Haviv, D. Purdie, D. Whiteman, N. Zeps, Anna DeFazio, David D. L. Bowtell, Elizabeth L. Christie

**Affiliations:** 1 Peter MacCallum Cancer Centre, Melbourne, VIC 3000 Australia; 2 Sir Peter MacCallum Department of Oncology, The University of Melbourne, Victoria, 3010 Australia; 3 Box Hill Hospital, Eastern Health, Box Hill, Victoria, 3128 Australia; 4 Centre for Cancer Research, The Westmead Institute for Medical Research, Sydney, NSW 2145 Australia; 5 The Daffodil Centre, The University of Sydney, a joint venture with Cancer Council NSW, Sydney, NSW 2006 Australia; 6 Department of Gynaecological Oncology, Westmead Hospital, Sydney, NSW 2145 Australia; 7 QIMR Berghofer Medical Research Institute, Brisbane, QLD 4006 Australia; 8 Department of Pathology, University of Melbourne, Victoria, 3010 Australia; 9 Sir Peter MacCallum Department of Oncology, The University of Melbourne, Victoria, 3010 Australia; 10 Department of Biochemistry and Molecular Biology, The University of Melbourne, Victoria, 3010 Australia; 11 Ovarian Cancer Action Research Centre, Department of Surgery and Cancer, Imperial College London, London, W12 0HS England; 12 The University of Sydney, Sydney, NSW 2006 Australia; 13 Melbourne School of Population and Global Health, The University of Melbourne, Victoria, 3010 Australia; 14 John Hunter Hospital, Lookout Road, New Lambton, NSW 2305 Australia; 15 Royal Hospital for Women, Barker Street, Randwick, NSW 2031 Australia; 16 Royal North Shore Hospital, Reserve Road, St Leonards, NSW 2065 Australia; 17 Royal Prince Alfred Hospital, Missenden Road, Camperdown, NSW 2050 Australia; 18 Royal Adelaide Hospital, North Terrace, Adelaide, South Australia 5000 Australia; 19 Royal Hobart Hospital, 48 Liverpool St, Hobart, TAS 7000 Australia; 20 Monash Medical Centre, 246 Clayton Rd, Clayton, VIC 3168 Australia; 21 Western Australian Research Tissue Network (WARTN), St John of God Pathology, 23 Walters Drive, Osborne Park, WA 6017 Australia; 22 Women and Infant’s Research Foundation, King Edward Memorial Hospital, 374 Bagot Road, Subiaco, WA 6008 Australia; 23 St John of God Hospital, 12 Salvado Rd, Subiaco, WA 6008 Australia; 24 Canberra Hospital, Australian Capitol Territory, Yamba Drive, Garran, 2605 Australia; 25 Bankstown Cancer Centre, Bankstown Hospital, 70 Eldridge Road, Bankstown, NSW 2200 Australia; 26 Northern Haematology & Oncology Group, Integrated Cancer Centre, 185 Fox Valley Road, Wahroonga, NSW 2076 Australia; 27 Illawarra Shoalhaven Local Health District, Wollongong Hospital, Level 4 Lawson House, Wollongong, NSW 2500 Australia; 28 Nepean Hospital, Derby Street, Kingswood, NSW 2747 Australia; 29 Newcastle Mater Misericordiae Hospital, Edith Street, Waratah, NSW 2298 Australia; 30 Port Macquarie Base Hospital, Wrights Road, Port Macquarie, NSW 2444 Australia; 31 Prince of Wales Clinical School, University of New South Wales, Sydney, NSW 2031 Australia; 32 St George Hospital, Gray Street, Kogarah, NSW 2217 Australia; 33 St Vincent’s Hospital, 390 Victoria Street, Darlinghurst, NSW 2010 Australia; 34 Wagga Wagga Base Hospital, Docker St, Wagga Wagga, NSW 2650 Australia; 35 Crown Princess Mary Cancer Centre, Westmead Hospital, Westmead, Sydney, NSW 2145 Australia; 36 Department of Pathology, Westmead Clinical School, Westmead Hospital, The University of Sydney, Camperdown, NSW 2006 Australia; 37 Mater Misericordiae Hospital, Raymond Terrace, South Brisbane, QLD 4101 Australia; 38 The Royal Brisbane and Women’s Hospital, Butterfield Street, Herston, QLD 4006 Australia; 39 Wesley Hospital, 451 Coronation Drive, Auchenflower, QLD 4066 Australia; 40 Burnside Hospital, 120 Kensington Road, Toorak Gardens, South Australia 5065 Australia; 41 Queen Elizabeth Hospital, 28 Woodville Road, Woodville South, South Australia 5011 Australia; 42 Freemasons Hospital, 20 Victoria Parade, East Melbourne, Victoria, 3002 Australia; 43 Mercy Hospital for Women, 163 Studley Road, Heidelberg, VIC 3084 Australia; 44 The Royal Women’s Hospital, Parkville, VIC 3052 Australia; 45 Border Medical Oncology, Wodonga, VIC 3690 Australia; 46 Andrew Love Cancer Centre, 70 Swanston Street, Geelong, VIC 3220 Australia; 47 Ballarat Base Hospital, Drummond Street North, Ballarat, VIC 3350 Australia; 48 Bendigo Health Care Group, 62 Lucan Street, Bendigo, VIC 3550 Australia; 49 Peninsula Health, 2 Hastings Road, Frankston, VIC 3199 Australia; 50 Mount Hospital, 150 Mounts Bay Road, Perth, WA 6000 Australia; 51 Faculty of Medicine, Bar-Ilan University, 8 Henrietta Szold St, Safed, Israel

**Keywords:** Cancer genomics, Ovarian cancer

## Abstract

While the introduction of poly-(ADP)-ribose polymerase (PARP) inhibitors in homologous recombination DNA repair (HR) deficient high grade serous ovarian, fallopian tube and primary peritoneal cancers (HGSC) has improved patient survival, resistance to PARP inhibitors frequently occurs. Preclinical and translational studies have identified multiple mechanisms of resistance; here we examined tumour samples collected from 26 women following treatment with PARP inhibitors as part of standard of care or their enrolment in clinical trials. Twenty-one had a germline or somatic *BRCA1/2* mutation. We performed targeted sequencing of 63 genes involved in DNA repair processes or implicated in ovarian cancer resistance. We found that just three individuals had a small-scale mutation as a definitive resistance mechanism detected, having reversion mutations, while six had potential mechanisms of resistance detected, with alterations related to *BRCA1* function and mutations in *SHLD2*. This study indicates that mutations in genes related to DNA repair are detected in a minority of HGSC patients as genetic mechanisms of resistance. Future research into resistance in HGSC should focus on copy number, transcriptional and epigenetic aberrations, and the contribution of the tumour microenvironment.

## Introduction

High grade serous ovarian, fallopian tube and primary peritoneal cancer (HGSC) is the archetypal homologous recombination DNA repair (HR)-deficient cancer, with a high frequency of germline or somatic *BRCA1/2* and other HR gene aberrations^1–3^. Genomic and epigenetic aberrations in these genes are now well accepted as predictors of survival and platinum sensitivity in HGSC; similarly, these factors also predict sensitivity to poly-ADP-ribose polymerase (PARP) inhibitors^2,4,5^. PARP inhibitors (PARPi) interfere with the enzymatic action of *PARP1* and *PARP2*, disrupting base excision repair, as well as trapping PARP on sites of DNA damage resulting in stalling of replication forks and ultimately double stranded breaks^6^. In HR deficient tumour cells, cells utilise error prone non-homologous end joining (NHEJ) to avoid an accumulation of unrepaired DNA damage^7,8^. Multiple successful clinical trials have led to PARP inhibitors now being firmly embedded as standard of care in the maintenance treatment of HR-deficient epithelial ovarian cancer^9–12^.

Despite impressive gains in progression-free and overall survival, and individual patients with exceptional responses, resistance to PARPi eventuates in most individuals with HGSC^10,13,14^. Secondary somatic mutations in HR genes such as *BRCA1, BRCA2, RAD51C, PALB2, *or* BRIP1*^15–18^, termed reversion mutations, fully or partially restore protein function and are one of the most prevalent and well-known causes of platinum and PARPi resistance^4,19,20^. Reversions have been identified in a range of cancer types in addition to epithelial ovarian cancer^21–23^. Recent data from prospective trials observed reversion mutations in 26% of patients with ovarian cancer progressing on olaparib^24^.

Far smaller numbers of patients have been identified with non-reversion mechanisms of PARP inhibitor resistance, such loss of *TP53BP1* or *MRE11* amplification^25^. Numerous additional PARPi resistance mechanisms have been identified in preclinical studies, including *PARP1* mutations, *TRIP13* overexpression, alternative splicing of *BRCA1,* and Shieldin complex inactivation^26–29^. However, few of these mechanisms have been observed in clinical samples, and there remains an incomplete understanding of the mechanisms of resistance to PARPi in HGSC patients. Therefore, we sought to investigate the frequency and nature of small-scale mutations as a mechanism of PARPi resistance in a cohort of 26 women with HGSC who had received a PARPi as part of their clinical care.

## Results

### Study cohort

**Figure 1:**
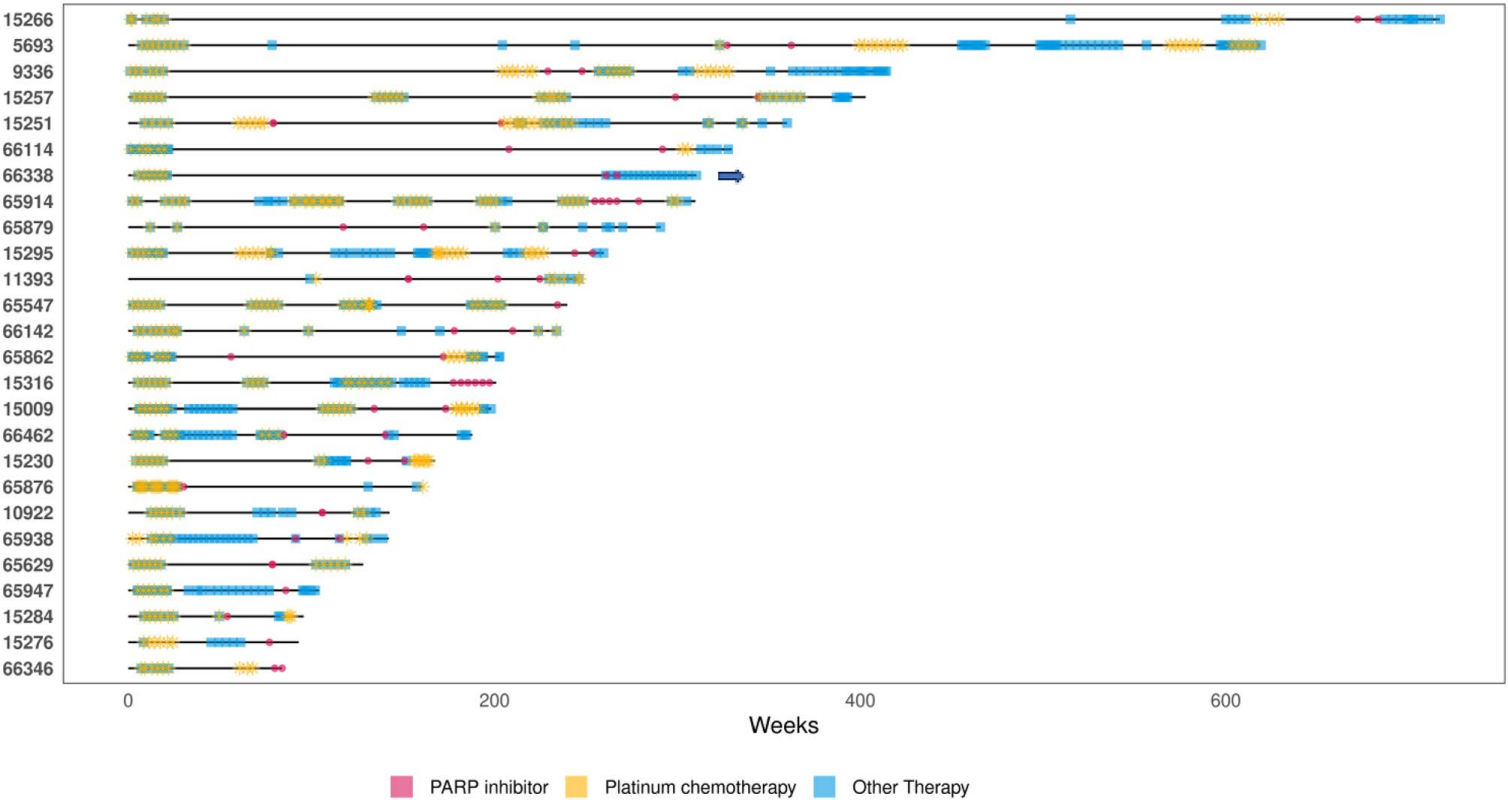
Cohort treatment overview. Swimmer plot showing the treatments received by the 26 HGSC patients in the cohort. Each asterisk represents a cycle of treatment or for PARPi the start, stop or cycle dates (see “Methods”), the colour of the symbol represents the type of treatment as indicated in the figure legend. The x-axis represents the timing of treatment in weeks from diagnosis. Arrow represents only living patient at time of data extraction.

We identified a cohort of 26 individuals with HGSC who were recruited to the Australian Ovarian Cancer Study (AOCS) between 2004 and 2018, had been treated with a PARPi and where a post-PARPi treatment tumour sample was available. These were taken either as part of clinical trials or as part of their standard of care treatment, and were variable with regard to timing between biopsy and last PARPi. Thirteen of the patients had a pre-PARPi sample available for comparison. The clinical characteristics of the cohort are summarised in Table 1. The patients received a median of 5 lines of treatment (Table 1, Fig. 1). We were able to access the exact dates of PARPi treatment for 19 of the patients, and they spent a median of 224 days receiving PARPi (range 28–873 days). While most patients had a known pathogenic germline or somatic *BRCA1/2* mutation, some patients were participants in clinical trials which did not require this as an inclusion criterion.

**Table 1:** Clinical cohort characteristics.

	Cohort n (%)
Age	
Median	55
Range	35–77
FIGO Stage	
IIIA	2 (7.7%)
IIIB	2 (7.7%)
IIIC	16 (62%)
IV	2 (7.7%)
Overall survival (weeks)	
Median	236
Range	84–717
Lines of therapy	
Median	5
Range	2–9
HR gene mutation	
Germline *BRCA1*	13
Germline *BRCA2*	4
Somatic *BRCA1*	2
Somatic *BRCA2*	1
Unknown *BRCA1*	1
None (wildtype)	5
	Total n = 26

#### Targeted sequencing identifies *BRCA1/2* and *TP53* mutations

A capture-based targeted sequencing panel was previously designed to analyse the exons of 63 genes (Supplementary Table 1) implicated in DNA repair or chemotherapy resistance in HGSC^18^. Targeted sequencing was performed on 39 tumour and 24 germline samples, with median coverage across all samples of 500X, and 96% of target bases achieved at least 100X coverage.

Seventeen patients had germline *BRCA1/2* mutations and 3 had somatic *BRCA1/2* mutations (Table 1, Supplementary Table 2). One patient had a *BRCA1* mutation detected in their tumour, however as a germline sample was not available, we were not able to determine whether this was a germline or somatic mutation, and a clinical germline testing result was not available. Sixteen of the patients had mutations in *BRCA1* and 5 were in *BRCA2*. Five patients had no detectable mutations in HR genes. One patient had the presence of a *BRCA1* mutation documented in their clinical notes but without cDNA or protein annotation; and no mutation was detected in this patient in the targeted sequencing.

We used the *TP53* mutation variant allele frequency (VAF) to infer tumour content, since *TP53* mutations are near ubiquitous and are early events in HGSC^30,31^. Twenty-four of the 26 post-PARP inhibitor samples had a *TP53* mutation identified, and 11 of thirteen pre-PARP inhibitor samples had the same corresponding *TP53* variant identified. When identified, the median *TP53* allele frequency was 63% (IQR 25–84%) (Supplementary Table 3).

#### Mechanisms of resistance

##### Reversion mutations

**Figure 2:**
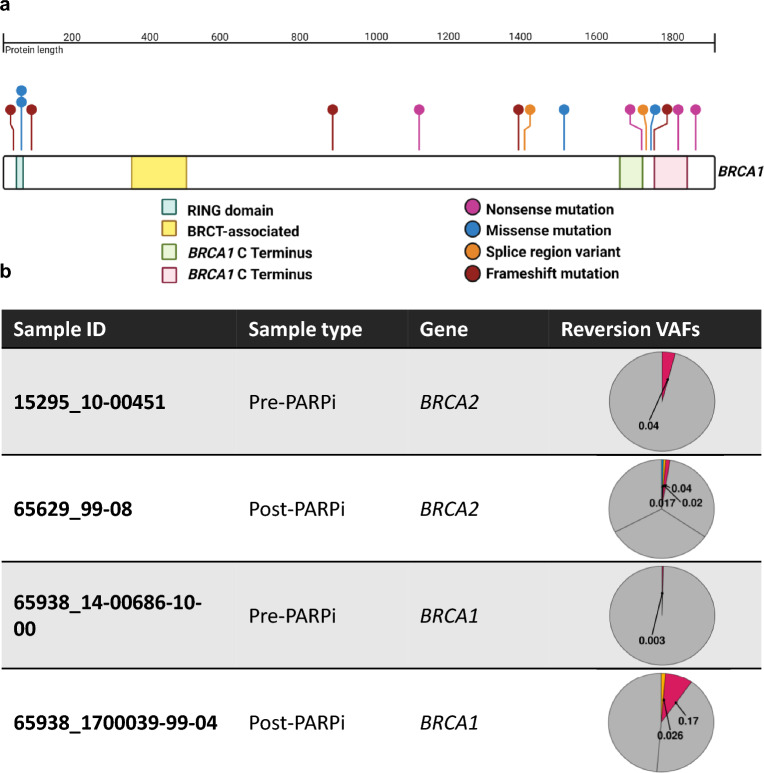
Schematics of the mutations in the cohort. (**a**) The position of each *BRCA1* mutation is represented in the lollipop plot, with the number of dots on the lollipop indicating the number of patients in which the mutation is observed, the colour of the bar and point indicates the type of mutation. The blue lollipop with 2 dots denotes the Cys61Gly mutation. Schematic constructed based on lollipop plot generated from GenomePaint^61^. Note only 15 of 16 mutations are depicted, as one was annotated in AOCS but not detected in our sequencing. (**b**) Reversion mutations detected in three patients, the variant allele frequencies depicted as pie charts. Note that for 15295_10-00451, one reversion is not shown as it is a large deletion and the VAF not accurately estimable.

Of the 21 patients with *BRCA1/2* mutations whereby a reversion could restore HR and cause treatment resistance, reversions were found in only three patients, and two of these were in post-PARP inhibitor samples (Fig. 2). The number of unique reversions ranged between 2 and 3 per patient. All of the reversions were present at low variant allele frequencies, from < 1%-17.0%. This suggested the reversions were subclonal, although this could not be confirmed as the targeted sequencing panel does not have sufficient off-target coverage to determine local copy number state and therefore the cancer cell fraction. Both 65938 and 15295 had potential evidence of a reversion in their pre-PARPi sample, which we have previously described^18^. Case 15295 had a reversion detected in her pre- but not post-PARPi ascites sample. Case 65629 had a germline mutation which falls into a known hotspot susceptible to reversion (c.771-775del; p.Asn257LysfsTer17)^32^.

##### Position of *BRCA1* mutations

The position of the pathogenic mutation in *BRCA1* has been reported to have an impact on both pathogenicity and HR function, and as such platinum and PARP inhibitor resistance^29,33,34^. Hence, individuals with *BRCA1* mutations were examined for the position of their variant and the potential contribution to resistance.

Cases 66338 and 5693 both had a germline *BRCA1* p.Cys61Gly pathogenic mutation. This mutation occurs in the Really Interesting New Gene (RING) domain (Fig. 2), which has been demonstrated in animal models to confer resistance to platinum and PARPi, even while it permits tumourigenesis^33,35^. Despite this, neither of these patients had an especially poor response to their treatment, with both patients having an overall survival of greater than 5 years (Fig. 1), which is similar to the expected survival of a patient with *BRCA1*-mutated HGSC^36,37^. Case 5693 received single agent PARP inhibitor for 8 months, and although she had a rising CA125 relatively soon after, she subsequently responded to platinum chemotherapy. Case 66338 received a PARPi in combination with atezolizumab, making interpretation of response to the PARPi more difficult to dissect, however she had a prompt CA125 response on commencement of therapy.

Four patients had a mutation immediately flanking or between the 2 *BRCA1* C Terminus (BRCT) regions. Mutations in this region can be affected by Alu-mediated rearrangements, which can lead to evasion from proteasomal degradation and thereby propagate PARPi resistance^38,39^. Because the sequencing panel was primarily designed to capture exons, coverage over intron 15 which would be required to assess for this mechanism of resistance was negligible. Therefore, we were not able to examine structural variants in this region, and this could be an undetected source of resistance for the tumours in these 4 patients.

##### Non-*BRCA1/2* mutations

Having assessed reversion mutations, we examined mutations in other genes for potential mechanisms of resistance. We manually reviewed 24 variants in post-PARPi tumour samples from 22 patients with a matched germline sample, and 109 variants in 2 patient samples without a germline sample. Specifically, these variants were reviewed for their likelihood to cause resistance based on the protein consequence, the Ensembl Variant effect Predictor SIFT and PolyPhen algorithm scores, and whether they have previously been described in literature as pathogenic. Six patients were found to have a mutation that was considered as a potential resistance mechanism; these were in 5 genes (Table 2).

**Table 2:** Mutations considered as potential resistance mechanisms.

Patient ID	Sample type	*BRCA1/2* mutation	Gene	Consequence	HGVSp	VAF	Present Pre-PARPi
65879	Post-PARPi	*BRCA1*	*ARID1A*	Stop gained	p.Gln878Ter	0.05	NA
15266	Post-PARPi	*BRCA2*	*CHEK2*	Missense variant	p.Arg160Gly	0.44	NA
15266	Post-PARPi	*BRCA2*	*XRCC3*	Missense variant	p.Thr241Met	0.48	NA
15257	Post-PARPi	*BRCA1*	*SHLD2*	Missense variant	p.Trp11Arg	0.05	NA
15276	Post-PARPi	*BRCA1*	*SHLD2*	Stop gained	p.Gln169Ter	0.37	No
15284	Post-PARPi	*BRCA1*	*SHLD2*	Missense variant	p.Lys438Glu	0.16	NA
15230	Post-PARPi	*BRCA1*	*TP53BP1*	Missense variant	p.Lys1141Gln	0.46	NA

The Shieldin complex, comprised of *SHLD1*, *SHLD2* and *SHLD3*, acts downstream of *TP53BP1*, *RIF1* and *REV7* to promote non-homologous end-joining. Hence, loss of function of the complex is expected to divert double stranded DNA break repair toward homologous recombination in the context of *BRCA1* loss^26,40^. Case 15276, who had a germline *BRCA1* mutation, was found to have a somatic truncating *SHLD2* mutation, which was not present in their pre-PARPi sample. It was noted that this patient had the second lowest survival in the cohort (Fig. 1). Two additional patients with germline *BRCA1* mutations, Cases 15284 and 15257, had a somatic missense variant identified in *SHLD2* (Table 2).

As for the Shieldin complex, loss of *TP53BP1* in a *BRCA1* mutant context promotes DNA repair by HR over NHEJ^41^. Case 15230 with *BRCA1*-mutated HGSC was found to have a missense *TP53BP1* p.Lys1141Gln variant at a VAF of 46% in the post-PARPi tumour sample. No pre-PARPi sample was available for this patient to determine the timing of development of this variant.

Other variants were more tenuous in their likelihood of contributing to PARPi resistance. These included a somatic *CHEK2* p.Arg160Gly variant in case 15266, who had a germline *BRCA2* mutation. This variant falls within the forkhead-associated domain of *CHEK2*, and has been suggested to contribute to an increased risk of development of breast and prostate cancer^42,43^. This patient did not have a pre-PARPi sample available to ascertain if the variant was acquired during PARPi treatment and it is unclear what the implication of this mutation might be for resistance, rather than cancer inception, particularly in the context of a germline *BRCA2* mutation. Case 15266 was also found to have a somatic missense *XRCC3* mutation. *XRCC3* is a paralog of *RAD51* and is involved in HR^44^. PARPi resistance via aberrant *XRCC3* has not been explicitly described in the literature, but since other *RAD51* paralogs have been demonstrated to contribute to resistance through gene silencing it is plausible that *XRCC3* could play a similar role^45^.

Finally, case 65879, who had a germline *BRCA1* mutation, had a somatic *ARID1A* truncating mutation detected. *ARID1A* is a critical subunit of the SWI/SNF complex mutated in many cancer types. It is recruited to sites of double stranded DNA damage via its interaction with *ATR*. *ARID1A* deficiency attenuates *ATR* activation and therefore end resection of DSBs, however conflicting consequences of loss of *ARID1A* gene function on response to PARP inhibition have been reported^46,47^. Thus, it is unclear if this loss of function mutation could plausibly confer PARPi resistance.

No structural variants (SVs) relevant to resistance were detected. Specifically, we did not find evidence of SVs in intron 1 of *ABCB1* that may lead to a transcriptional fusion, which have previously been described as a mechanism of resistance in HGSC for P-glycoprotein substrates (including several chemotherapies and PARPi)^48^, neither did we observe SVs that could be reversion mutations.

## Discussion

In this study we used a targeted DNA sequencing approach to identify mechanisms of resistance to PARPi in HSGC via small-scale mutations, through analysis of tumour samples collected from individuals with HGSC after completion of PARPi treatment. We identified a definitive resistance mechanism in only three of the 26 cases, with an additional six cases with variants potentially contributing to resistance, and a further three with variants in genes that less clearly contribute to resistance. All cases with reversion mutations had more than one unique reversion detected. Aside from reversion mutations, only one case had more than one variant that may be a mechanism of resistance identified.

Reversions were identified in 14% of the cohort, which is a similar frequency to that seen in other studies^21,24,49,50^. For example, consistent with our findings, Lin et al. reported reversions in 13% of platinum resistant tumours, however others have reported higher frequencies of 26–41% in similar contexts^21,24,49,50^. That the prevalence of reversion mutations in our study were on the lower end of the spectrum of that previously observed may reflect that there were more tumours from this cohort associated with *BRCA1* mutations rather than *BRCA2*, as previous studies have shown a trend (albeit not statistically significant) towards reversions being more prevalent in *BRCA2* mutant tumours^18,21^. Sample type, timing of sample collection relative to prior treatment and progression, and sequencing methodology varies across studies, all of which may also affect the frequency of reversion detection. The three patients with reversions all had a varying CA125 response to PARP inhibition; 2 of these already had reversions prior to receiving PARPi as described previously^18^.

Many of the preclinical, non-reversion mechanisms of resistance described in the literature are specific to *BRCA1*, for example loss of expression of the Shieldin complex^26,40^, therefore it may be that tumours associated with *BRCA2* alterations have fewer pathways to resistance and therefore reversions predominate. The identification of three cases with germline *BRCA1* mutations with somatic *SHLD2* mutations in post-PARPi tumour samples suggests that through loss of the Shieldin complex, cells are diverted from NHEJ, restoring homologous recombination, leading to resistance. The position of a mutation within the *BRCA1* gene has also been associated with response to PARP inhibitors, with the largest analysis of this in a post-hoc analysis of PAOLA-1^34^. However, as others have observed, the exact relationship between mutation position and clinical outcome is likely to be nuanced^33^, and the cases in our cohort with mutations in the RING domain did not have an especially poor response to treatment.

Resistance is likely to occur at a subclonal level, as reported by many others^51–53^, and it is possible that some variants could be present at very low levels. With a median of 500X coverage, however, the likelihood of missing variants present at a sufficiently high frequency to impact clinical response is relatively low. Finally, when this project was conceived, there were limited studies describing the frequency of genetic mechanisms of PARPi resistance, with the exception of reversion mutations. Recent and complementary work made concordant findings, that genetic mechanisms of resistance in HGSC aside from reversions are uncommon^54,55^.

As our work focussed on a list of curated genes specifically relevant to DNA repair and ovarian cancer resistance it is possible that resistance causing mutations in genes not represented on the panel have been missed. Countering this view, other studies using more general cancer panels have not detected large numbers of non-reversion resistance mechanisms^4^. Additionally, our targeted sequencing panel has insufficient off-target reads to examine somatic copy number aberrations. As others have noted, the copy number landscape of HGSC appears relatively stable between diagnosis and relapse^56^, however this has not to our knowledge been examined specifically with regard to PARP inhibitor receipt.

In conclusion, this study adds further evidence to the growing notion that small-scale mutations as a mechanism of resistance in genes implicated in DNA repair mechanisms are not common in HGSC. This suggests the majority of PARPi-mediated resistance may be occurring at a copy number, transcriptional, epigenetic or immune microenvironmental level, and that future work should focus on these aspects. Large datasets will be required to have sufficient statistical power to robustly identify novel mechanisms. Finally, intra-patient heterogeneity does not only occur at a DNA level, and the subclonality of resistance mechanisms represents a challenge for the design of treatments to counter resistance that may be detected in the future.

## Methods

### Cohort

Ethics approval was obtained for access to clinical data and analysis of samples collected by the Australian Ovarian Cancer Study (AOCS) (Peter MacCallum Cancer Centre HREC no. 15/84), and all methods were performed in accordance with this approval and within institutional guidelines. Written informed consent was obtained from all participants in this study by AOCS.

The AOCS database was interrogated to identify participants with HGSC who had received a PARP inhibitor, and who had a post-PARPi ascites, pleural effusion, or solid snap frozen tumour sample available for research. Additionally, for inclusion in this study either a germline or pre-PARPi tumour sample was required for comparison, and sufficient clinical information to assess patient response to PARP inhibitor treatment. The details of PARPi receipt were recorded inconsistently in the medical record and therefore also in the AOCS data (for example, some were recorded as a start and stop date, others as cycles, and others with only an inferred stop date); so as not to introduce any assumptions, these descriptors were retained and have been reported verbatim. Additionally, types of PARPi were variably documented either as the drug name or sometimes simply as ‘PARP inhibitor’, hence individual PARPi type is not reported here.

#### Tissue processing, nucleic acid isolation and DNA sequencing

As described previously, AOCS processed ascites and pleural effusions to isolate tumour cells, and DNA was isolated from germline (peripheral lymphocytes) and tumour samples using the salting out method and the DNeasy blood and tissue kit (QIAGEN), respectively^49^. The targeted hybrid capture panel, as described in Burdett et al.^18^, comprised 63 genes implicated in homologous recombination and alternative DNA repair mechanisms, or chemotherapy resistance in ovarian cancer (Design no. 3221041). Briefly, 120 ng of germline or tumour DNA was used as input for library preparation using the Agilent SureSelect Low Input Target Enrichment System as per the manufacturer’s protocol. Libraries were sequenced using the Illumina NextSeq 500 at the Peter MacCallum Cancer Centre.

#### Sequencing data processing

FASTQ files were aligned to reference genome version hg19. Variant calling for point mutations, INDELs and structural variants was performed as per our established pipeline^18^. Briefly, four variant callers for SNV/indels were employed (VarDictJava (v1.5.7), Strelka2 (v2.9.9), Varscan2 (v2.4.3) and Mutect2 (v4.0.11.0)^57–59^) to generate consensus variant calls for those with a germline sample, and for the 2 samples where there was no germline sample, only VarDictJava was run. GRIDSS was used to look for structural variants^60^.

#### Post-processing data analysis

In the cases with a germline sample (n = 24), there were 823 high confidence, non-synonymous mutations identified by consensus variant call. The two cases without a matched germline sample had 690 high confidence, non-synonymous variants detected, due to the lack of a germline sample with which to filter out inconsequential single nucleotide polymorphisms.

Manual curation of *TP53* and HR variants was performed in IGV v2.11.9 and the web-based platform. Reversions were manually reviewed and were confirmed if they occurred in the same reads as the germline or somatic mutation. Reversions were classified as:

- High confidence: > 10 reads or > 5% VAF
- Moderate confidence: > 5–10 reads
- Low confidence: ≥ 2–5 reads

Other consensus variants (≥ 2 variant callers) that were called in post-PARPi samples were assessed as described in Results. All analyses and statistics were performed in RStudio v4.1.0. Figures were generated in RStudio, BioRender.com, and the *BRCA1* schematic was constructed based on the lollipop plot generated using GenomePaint^61^.

### Supplementary Information

Supplementary Information.

## Data Availability

Targeted capture sequencing FASTQ files for each sample type (tumor/normal) is being deposited in the EGA repository under accession code EGA [TBA] (https://ega-archive.org/studies/). Information on how to apply for access is available at the EGA under accession code EGA [TBA].
